# Lifestyle, Insulin Resistance and Semen Quality as Co-Dependent Factors of Male Infertility

**DOI:** 10.3390/ijerph20010732

**Published:** 2022-12-30

**Authors:** Adrianna Zańko, Katarzyna Siewko, Adam Jacek Krętowski, Robert Milewski

**Affiliations:** 1 Doctoral School, Medical University of Białystok, 15-089 Białystok, Poland; 2 Department of Endocrinology, Diabetology and Internal Medicine, Medical University of Białystok, 15-276 Białystok, Poland; 3 Department of Biostatistics and Medical Informatics, Medical University of Białystok, 15-295 Białystok, Poland

**Keywords:** fertility, insulin resistance, semen quality, lifestyle

## Abstract

Infertility is a problem that affects millions of couples around the world. It is known as a disease of couples, not individuals, which makes diagnosis difficult and treatment unclear. Male infertility can have many causes, from mechanical ones to abnormal spermatogenesis or spermiogenesis. Semen quality is determined by a number of factors, including those dependent on men themselves, with the number of infertile men growing every year. These include, e.g., diet, physical activity, sleep quality, stress, among many others. As these factors co-exist with insulin resistance, which is a disease closely related to lifestyle, it has been singled out in the study due to its role in affecting semen quality. In order to examine connections between lifestyle, insulin resistance, and semen quality, a review of literature published from 1989 to 2020 in the following databases PubMed/Medline, EMBASE (Elsevier), Scopus, Web of Science, and Google Scholar was performed. Hence, semen quality, environment, and insulin resistance are interrelated, thus it is difficult to indicate which aspect is the cause and which is the effect in a particular relationship and the nature of possible correlations. Since the influence of lifestyle on semen quality has been extensively studied, it is recommended that more thorough research be done on the relationship between insulin resistance and semen quality, comparing the semen quality of men with and without insulin resistance.

## 1. Introduction

Infertility afflicts approx. 60–80 million couples worldwide [1]. As the condition affects couples, as opposed to individuals, they are the focus of diagnostics performed to determine the underlying causes. Male infertility may result from spermatogenesis or spermiogenesis failure, as well as from the existence of mechanical obstructions that prevent healthy sperm from fusing with the ovum [2]. It is recommended that semen examination should be performed after sexual abstinence of 2–7 days, with its normal parameters determined by the WHO. Analyses of sperm parameters should be performed manually by specialized diagnosticians or with the use of the computer methods enabling a very accurate assessment of sperm motility in the seminal fluid, e.g., CASA (Computer Assisted Sperm Analysis). Sperm parameters as examined by WHO are: sperm count, sperm motility, morphology, progressive motile sperm count, pH, and semen volume [3]. The quality of semen is influenced by numerous factors, some of which can be influenced, ranging from genetics or diseases to lifestyle and its components, such as diet, physical activity, or sleep quality, among many others. A metabolic disorder closely connected with lifestyle is insulin resistance [4], which seems to be a highly significant factor in infertility diagnostics as it affects most of those components of lifestyle that have an impact on the quality of semen. Hence, the aim of this review was to determine whether performing insulin resistance tests in the context of infertility should become a new diagnostic standard in view of its role in causing male infertility.

## 2. Sources of Articles, Search Strategies, and the Selection Process

### 2.1. Sources of Information

The study was carried out as a systematic review of literature published from 1989 to 2020 in the following databases: PubMed/Medline, EMBASE (Elsevier), Scopus, Web of Science, and Google Scholar.

### 2.2. Search Strategy for the Identification of Studies

The search strategy was discussed with researchers familiar with the topic, employees of the administering institute. The selected electronic databases were open access. The following search terms were used: “fertility”, “men fertility”, “infertility”, “lifestyle and fertility”, “insulin resistance and fertility”, and “insulin resistance in men”. The keywords were selected on the basis of their relevance to the topic and commonality of use; they were also adapted to each of the databases.

### 2.3. Inclusion/Exclusion Criteria: Data Management and Study Selection

The data was examined by two independent reviewers and included information, such as: authors, title, journal, and year. Titles and abstracts were previewed in order to identify potentially relevant/irrelevant articles. The results were entered into an Excel spreadsheet; the articles were marked as “included” or “excluded”, with an explanation for the reason for exclusion. All disagreements between the researchers were resolved by consensus, with a third researcher engaged as needed.

**Figure 1:**
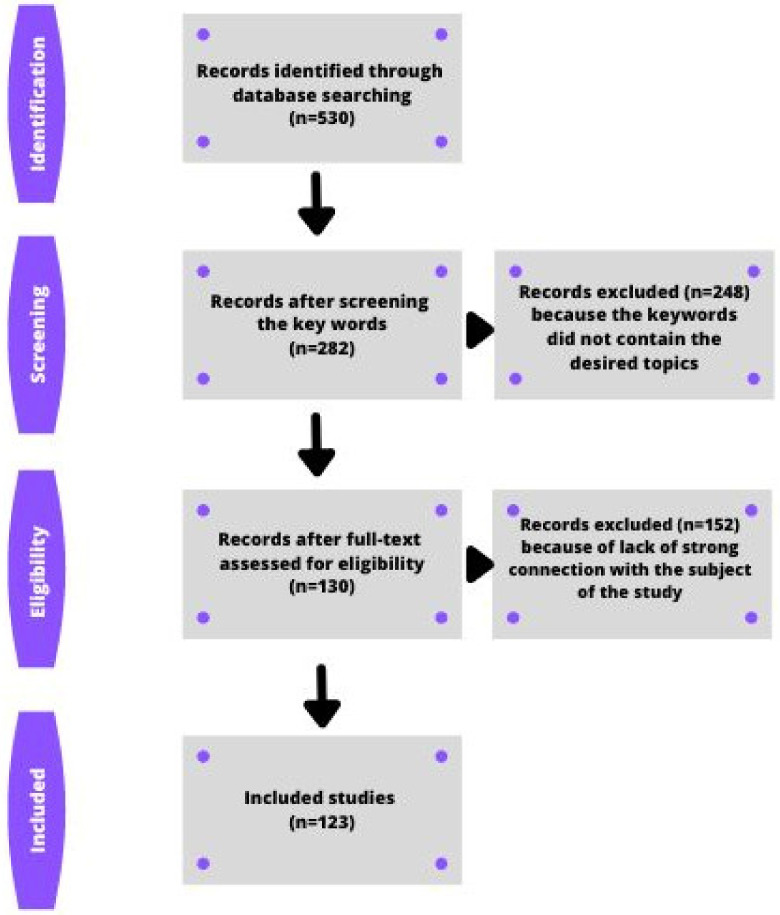
Flow-chart explaining the process of selecting articles.

The process of inclusion and exclusion of articles was divided into the following four stages: ‘identification’, ‘screening’, ‘eligibility’, and ‘inclusion’. During identification, 530 articles were considered, with 248 of them excluded at the screening stage due to a lack of relevant keywords. At the eligibility stage, a further 152 papers were excluded due to a lack of sufficient relevance to the topic. At the inclusion stage, 123 papers were ultimately included in the study, i.e., those that contained the relevant keywords; were relevant to the topic; and reflected the current scientific trends in the research area in question. In addition, each of the analyzed articles was checked by a person competent in the particular field of science as to its quality and lack of bias (Figure 1).

### 2.4. Data Extraction

The analyzed data included: the authors’ names, the year of publication, the country, the research aims, the sample size, the participants, and a summary of results.

### 2.5. Quality Assessment of Studies

Each article included in the study was analyzed in terms of its methodological quality by two independent researchers competent in the topic of semen quality and insulin resistance. The researchers paid attention to the reliability, bias, and relevance of data to the analyzed topic.

## 3. Lifestyle and Insulin Resistance

It has been known for a long time that lifestyle influences the functioning of the whole body—decreasing or increasing the risk of diseases, weakening or strengthening the body. The quality of semen is no exception, i.e., physical, chemical, or biological factors originating from the external environment, in addition to those connected with lifestyle, may either have a positive or a negative impact on the quality of semen [5]. Moreover, lifestyle may be responsible for sperm DNA damage [5].

Insulin resistance is a metabolic disorder and it is defined as the inability of a known quantity of insulin (exogenous or endogenous) to increase glucose uptake and utilization in an individual as much as it does in the healthy population [6]. Untreated insulin resistance leads to diabetes, with insulin resistant people needing larger amounts of insulin to transport glucose into their cells [7]. If not corrected through optimizing body weight, physical activity, and/or medication, insulin resistance often contributes to type 2 diabetes [8].

Insulin resistance among men is often connected with the metabolic syndrome and obesity, recognized as risk factors of decreased sperm quality [4,9]. Both this diseases are connected with impaired lipoprotein concentration [4]. As far as lipoprotein metabolism is concerned, there are studies showing correlations between insulin resistance and elevated triglyceride levels and reduced HDL [10]. Due to the fact that insulin resistance is increasingly often being diagnosed in the population, it demands a closer scrutiny in the context of semen quality. As the disorder is closely connected with lifestyle and civilization diseases, such as obesity and metabolic disorder [4], insulin resistance—as the underlying cause of diabetes—needs to be focused on due to its diagnostic potential in terms of male infertility.

Insulin resistance causes inflammation in the body, lowers sex hormone binding globulin (SHBG) levels in obese men, may affect the secretion of male sex hormones, and further exacerbates obesity, which is known to have a negative impact on the quality of semen [4,11,12,13].

Metabolic effects of insulin resistance in terms of sperm quality are not fully understood. It is known from scientific research that ejaculated sperm secretes insulin that controls the use of glucose in the sperm, which may affect its capacitation [14]. In addition, a study by Cappelo et al. identified a mitochondrial citrate carrier that contributes, through insulin secretion control, to the acquisition of fertilizing ability. This may mean that insulin has a complex role in sperm metabolism [15].

Few studies have been performed so far that describe the quality of semen in insulin resistance. The most commonly used methods to assess insulin resistance are indirect ones, consisting in the determination of fasting glucose and insulin concentrations, or in performing the oral glucose tolerance test. The most common method used in practice is measuring the HOMA-IR indicator (homeostasis model of assessment of insulin resistance), calculated on the basis of fasting insulin and glucose concentrations [16]. Ma et al. performed a study in which the levels of sex hormones were measured in the following three groups: T1 (HOMA-IR 0.36–0.55, n = 27), T2 (HOMA-IR 0.56–0.80, n = 28), and T3 (HOMA-IR 0.81–1.97, n = 28). The results showed that HOMA-IR was negatively correlated with serum testosterone level, semen volume, and the percentage of progressively motile sperm [6]. There was a negative correlation between insulin concentrations, SHBG, and testosterone concentrations in a study by Betancourt-Albrecht and Cunningham concerning prostate cancer [17]. There is also reason to suspect that the lack of insulin stimulation of Leydig cells, which may result from the insulin resistance of cells, may be the cause of reduced testosterone production [13].

In addition to the physiological and metabolic aspects, the impact of insulin resistance on mental well-being is worth mentioning. Insulin resistant persons often experience a depressed mood resulting from neurological disorders and fluctuations of glucose and insulin concentrations at the neural level [18]. A depressed mood may lead to decreased libido, worse dietary choices, or resorting to using psychoactive substances—which have an impact on the quality of semen [19,20].

Researchers show that DNA damage assessment may help to understand the epidemiology of male infertility [21]. Based on this assumption, it would be worth determining which components of lifestyle and which elements of the external environment may cause DNA damage and affect the quality of semen. Doubtlessly, these components include the following: diet, physical activity, sleep quality, stress, duration of sexual abstinence, and pollution from the external environment—all connected with insulin resistance.

### 3.1. Diet

Nutrients delivered to the body build its tissues and regulate the processes that take place in it. Individual nutrients as well as dietary patterns as a whole may have an impact on the quality of semen and male fertility [22,23,24]. As far as insulin resistance is concerned, nutrition is a key element of therapy. In terms of the diet of people insulin resistance, it is important that they eat regularly in addition to maintaining the proper the composition and volume of meals [25]. The main dietary recommendations aimed at preventing insulin resistance include the following: eating plant-based fiber sourced from products, such as oats, beans, peas, lentils, grains, seeds, fruits and vegetables, whole grain bread, brown rice, and pasta; daily consumption of at least five servings of fruit and vegetables; limiting the consumption of fat (especially saturated fatty acids); limiting the consumption of fried foods, sweetened drinks, and sweets as well as foods rich in fat and sugar; eating breakfast; controlling the portion size of meals and snacks, and the frequency of eating; limiting alcohol consumption [26].

Studies often compare the “healthy” diet with the “Western” diet. Gaskins et al. equates the “Western” diet with the consumption of processed meat products, butter, dairy, refined grains and sweets, and a low consumption of vegetables and fruit. The “healthy” diet, on the other hand, which the authors describe as “prudent”, is characterized by a high consumption of vegetables and fruit, fish and poultry, soy, low-fat dairy products, oils, and whole grain products. The results of the study show that men who keep the “moderate” diet exhibit greater sperm motility [27]. In their study, Oostingh et al. showed a positive correlation between healthy dietary patterns and sperm concentration, its motility, and total count [28]. A study performed by Liu et al. connected the “Western” dietary style with a reduced sperm concentration and worse morphology [29].

Epidemiological studies indicate that the consumption of saturated fats is associated with the presence of markers of insulin resistance [30,31]. Moreover, some studies concerning the quality of semen also focused on particular groups of food products. These indicate a correlation between the frequency of consumption of processed meat products and a lower total sperm count, worse sperm morphology, lower semen volume, and a lower percentage of progressive motile sperm [32,33]. Different conclusions were drawn by Maldonado-Carceles et al., who showed that in all probability, meat consumption does not have an impact on the quality of semen; the frequency of consumption of offal, however, was correlated with lower sperm motility, while the frequency of consumption of crustaceans was positively correlated with sperm motility [34]. Moreover, studies show that the consumption of saturated fatty acids (SFA) and trans fatty acids has an adverse effect on the quality of semen. SFA consumption is negatively correlated with the total sperm count and concentration, while the consumption of trans fatty acids is negatively correlated with total sperm count [35,36,37].

A study by Frazini et al. it showed that the proper intake of antioxidants sourced from diet may have a positive effect on glucose metabolism in the context of insulin resistance [38]. In addition, consuming omega-3 fatty acids derived from fish can also be helpful in this area [39]. Anti-inflammatory components of diet, such as omega-3 fatty acids or antioxidants have been known for a long time as ingredients that improve fertility in males [40,41,42]. A study by Jensen et al. showed that a higher consumption of omega-3 acids by men was connected with a higher semen volume [35], while a study by Attaman et al. showed a connection with a higher proportion of morphologically normal sperm [36]. In addition, Eslamian et al. showed that an increased consumption of omega-3 acids and DHA acid is connected with a lower risk of asthenozoospermia [43]. As far as antioxidants are concerned, they are mainly found in vegetables and fruit, as well as nuts and plant oils. A beneficial influence of vegetables and fruit on the quality of semen was shown in studies by Eslamian et al. and Mendiola et al., who proved that the consumption of these products is connected with a lower risk of asthenozoospermia and teratozoospermia [43,44].

Some scientists also focus on the assessment of particular diets that may improve the quality of semen. The Mediterranean diet is one of those that could be recommended in the context of the lifestyle of men with reduced semen quality, owing to the large contents of vegetables and unsaturated fatty acids, as well as fish. A positive effect of the Mediterranean diet on the quality of semen was shown in studies performed by Karayiannis et al., which indicated a relationship between the diet in question and increased sperm concentration, total sperm count, and progressive motile sperm count [45]. In their study, Cullitas-Tolin et al. also found a positive relationship between following the Mediterranean diet and an increased total sperm count [46]. Another diet worth considering is the DASH diet. Initially created for people with hypertension or at risk for it, it turns out to have a positive impact on the quality of semen [47]. In their study, Cullitas-Tolin et al. showed a positive relationship between the value of the DASH indicator and sperm concentration, total sperm count, and total progressive motile sperm count [46]. Other studies also show a positive correlation between the DASH indicator and higher normal sperm counts as well as the impact of the DASH diet on the increase of the antioxidative potential, which may alleviate changes in semen resulting from oxidative stress [48,49,50]. It needs to be emphasized that both the DASH diet and the Mediterranean diet are also indicated as the most beneficial ones in terms of the treatment and prevention of insulin resistance [51,52,53,54]. The use of the Mediterranean diet was associated with lower HOMA-IR values, lower insulin levels, and greater insulin sensitivity [52]. The DASH diet combined with a change of lifestyle showed improvement of insulin sensitivity [55].

Moreover, certain scientific analyses suggest an particularly beneficial effect of the individual components of diet on the quality of semen, e.g., zinc [56], vitamin B12 [57], or antioxidants [58]. In their paper, Boisen et al. formulated the hypothesis that vitamin D supplementation in men with semen quality disorders could be helpful due to its role in the production of male sex hormones [59]. Vitamin D deficiency, aside from its role incausing poor production of male hormones, may also increase the risk of insulin resistance, as it prevents the epigenetic changes that are associated with the disorder. Clinical trials indicate that vitamin D reduces insulin resistance and its effects [60]. Other studies indicate that supplementation with zinc and folic acid improves the quality of semen by increasing the total normal sperm count [61]. However, it is difficult to precisely determine which nutrients have a positive impact on the quality of male semen and whose impact is negative as this depends on the levels of micro- and macroelements in the body. Hence, both deficiencies and excessive levels of particular nutrients may have a negative impact on the quality of semen.

### 3.2. BMI

Studies of the quality of semen often determine the patient’s Body Mass Index (BMI). This is the most basic indicator used in research to divide the study group according to the participants’ anthropometric characteristics. Eisenberg et al. showed a positive correlation between body mass and abnormal semen volume and concentration. In this study Only 6% of men with normal BMI were oligospermic, and in the group of obese men—17% were oligospermic [62]. When studying the impact of body mass on the quality of semen, Hammiche et al. found a negative impact of overweight on semen volume, concentration, and total progressive motile sperm count [63]. Fariello et al., on the other hand, determined that among overweight people, a larger percentage of DNA was damaged, in comparison to the control group [64].

Obesity, as determined by BMI, is often connected with insulin resistance. The body composition indicators that determine obesity, such as high percentage of body fat, waist circumference, and subcutaneous adipose tissue content, as well as the incidence of the metabolic syndrome in men, studied by Gobato et al., correlated with the occurrence of insulin resistance [4]. Thus, it is unsurprising that in studies performed in children, obesity and insulin resistance are described as factors that significantly increase the risk of metabolic diseases in adulthood [65].

### 3.3. Physical Activity

Data concerning physical activity in the context of its effect on the quality of semen is conflicting. Although some studies indicate that it has no impact [66], most confirm a beneficial influence of recreational physical activity on the quality of semen both among healthy and infertile men [67,68,69,70]. For example, Danielewicz et al. found a positive correlation between the level of physical activity and sperm count (ΔT3-T1 = 69.4 mln/ej, ptrend = 0.043), progressive motile sperm count (ΔT3-T1 = 8.5%, ptrend < 0.001), and morphologically normal sperm count (ΔT3-T1 = 2.8%, ptrend = 0.003). In addition, coupled with the DASH diet, physical activity positively correlated with sperm concentration, total sperm count, progressive motile sperm count, and morphologically normal sperm count [22].

Scientists also focused on studying particular sport disciplines, or a particular type of physical activity. In their studies, Maleki and Tartibian showed that resistance exercises have a positive effect on reducing inflammation in the body as well as the level of oxidative stress, which correlates with improved semen quality and reduced percentage of damaged DNA [70]. In a study by Gaskins et al., weight training and open air exercises were proved to be beneficial for sperm concentration [68]. It is possible that the impact of weight training on improved seminological results was due to the fact that a moderately intense strength training influences the level of testosterone shortly after exercise and plays a role in reducing insulin resistance in healthy adult men [71].

Other studies showed a negative effect of a particular physical activity on the quality of semen—such were the conclusions of studies on the influence of cycling on the quality of semen [67,72]. Excessive physical effort, regardless of the discipline, seems to have a negative impact on the quality of semen [67]. This may be connected with the antioxidative capacities of the body, which can be reduced in men who train intensively, in comparison to men who train for recreation [70].

Physical activity is one of the factors that reduces insulin resistance. Studies performed in overweight or obese children and adolescents showed a positive effect of cardio training, particularly aerobic, on insulin resistance. Such exercises reduced both fasting insulin levels and the HOMA indicator values [71]. Similar relationships were found in studies in adults, i.e., after 8 weeks of aerobic exercise, reduced levels of fasting glucose and insulin were found in type 2 diabetes patients [73]. Hence, by treating the effects of metabolic disorders or preventing them—through the introduction of regular physical activity as early as at a young age—the quality of semen in the population can be improved.

### 3.4. Sleep

Sleep, as the state when our bodies regenerate, also seems to have an effect on the quality of semen. Poor sleep quality is observed particularly among shift workers, who disrupt their natural circadian rhythm, and usually sleep in short several hour-long segments [74]. A study by Chen et al. showed that too much or too little sleep, as well as poor sleep quality, affect semen quality. According to the researchers, the optimum sleep duration beneficial for the quality of semen is 8–8.5 h [75]. Similar conclusions were drawn by Vigano et al., whose study showed a reduced semen volume in the case of men who had problems with falling asleep, with obesity and tobacco smoking as additional factors worsening the results [76]. A study by Du et al. is also worth mentioning—it showed that poor sleep quality in men was connected with lower total motility, concentration, progressive motility, morphologically normal sperm, and total sperm count [77]. This may be connected with melatonin concentrations in the body, whose urine levels correlate positively with sperm concentration and cellular oxidative DNA damage repair capacity [78].

Another phenomenon that often coexists with obesity is sleep apnea. It also has an impact on the quality of semen. Studies show that the incidence of sleep apnea correlates negatively both with testosterone concentration and erectile dysfunction. Sleep apnea is obviously connected with obesity, but its negative impact on the quality of semen is also thought to be connected with poor sleep quality, which disrupts the nocturnal testosterone rhythm [79].

Many hormones and enzymes are secreted in the diurnal rhythm, with the process engaging circadian and homeostatic mechanisms. A badly slept night may disrupt the secretion of hormones, such as cortisol or growth hormone (GH), which influence insulin secretion. With insufficient sleep, increased GH secretion is observed, contributing to a reduced glucose uptake in muscles, which in turn leads to insulin resistance [80]. Thus, sleep hygiene seems to be important both for fertility improvement and prevention of metabolic disorders.

### 3.5. Stress

Stress causes many negative processes in the body yet our health is strongly connected with it [81]. Mental stress causes disorders of secretion of hormones responsible for male fertility, thus lowering the quality of semen [78]. This influences semen quality via neuroendocrine factors, i.e., an increased level of glucocorticoids leads to decreased rates of testosterone excretion from Leydig cells, which impairs spermatogenesis [82]. It also causes oxidative stress in the body, in addition to exacerbating insulin resistance and inducing the proinflammatory cytokine cascade—with all these aspects having a negative impact on the quality of semen [83,84].

A study by Zou et al. showed that high levels of work-related stress have a negative effect on semen parameters, such as sperm concentration and total sperm count. The study also confirmed that the negative impact on the quality of semen is not observed in men who experienced high levels of social support as a means of alleviating stress [85]. As far as everyday stress is concerned, similar conclusions were drawn by Janevic et al., whose study connected perceived stress with reduced sperm motility, sperm concentration, and the percentage of morphologically normal sperm [86].

Everyday stress is not the only type that can influence the quality of semen. Studies show that persons exposed to sudden, intense stress also have reduced semen parameters [87,88]. For instance, some of the parameters of semen of men rescued from the 2008 Wenchuan earthquake were disrupted as late as 3 years after the event. According to the researchers conducting the study, this could be related to the post-traumatic stress disorder caused by the loss of a close person or property [88].

Furthermore, stress is also extremely destructive for people with insulin resistance. By acting through the hypothalamic–pituitary–gonadal axis and producing inflammation in the body, mental stress deregulates glucose metabolism and insulin secretion. Studying the differences in the incidence of insulin resistance among the white and black populations, Fuller-Rowler et al. discovered that stress is the main reason for the differences between the tested groups, rather than the genetic makeup [89]. From the point of view of fertility and the quality of semen, both due to its primary action as a mental problem and through its role in the development of metabolic disorders, stress is a cause of infertility that is among the most difficult ones to overcome.

### 3.6. Psychoactive Substances

A common issue related to attempts at reducing stress are psychoactive substances. Alcohol and cigarettes have been studied numerous times in terms of their influence on the quality of semen [90,91]. As far as tobacco is concerned, studies unanimously confirm that it has a negative impact on semen volume, sperm concentration, sperm motility, morphology, and total sperm count, as well as the levels of follicle stimulating hormone and free testosterone [90,92]. Men who smoke cigarettes are also at risk of increased fragmentation of semen DNA [93]. In addition, tobacco smoking increases the risk of insulin resistance and hyperinsulinemia, thus increasing the risk of cardiovascular diseases [94].

In terms of alcohol, it reduces morphologically normal sperm count and causes sperm DNA damage [92,95]. Moreover, certain studies show its negative impact on fertility [96,97]. Moving beyond the direct impact of the aforementioned psychoactive substances on the quality of semen, it should be remembered that both excessive tobacco smoking and alcohol consumption contributes to an increased secretion of Reactive Oxygen Species (ROS)—inducing inflammation in the body and destroying the balance between ROS and antioxidants [94,95,98].

### 3.7. Sexual Abstinence

As mentioned, the duration of sexual abstinence prior to the examination of semen quality, as recommended by the WHO, is 2 to 7 days [3]. It is difficult to determine the ideal duration of sexual abstinence for optimum results of semen quality assessments due to the fact that some of its parameters improve while others worsen over time [99].

In the case of semen volume and sperm count, the following regularity can be noted: the longer the abstinence, the higher the levels of these parameters, with a particular increase in their values occurring after over 5 days of abstinence [100]. In the case of parameters, such as morphology, motility, and DNA fragmentation, it seems true that they improve after a shorter abstinence time (up to 4 days) [99].

Among men with insulin resistance, particularly in the group of obese persons, sexual abstinence is sometimes prolonged. This results from decreased libido, caused by deficiencies in sex hormones, especially testosterone, as well as abnormal androgen-estrogen ratios [101]. This fact also constituted a psychological burden; hence, it is again connected with an increased risk of semen quality disorders and infertility.

### 3.8. External Environment

Care for the natural environment also helps to maintain health. Pollutants are regularly emitted to the atmosphere, soil, or water, entering our bodies through the respiratory and the digestive systems [102]. Scientific studies examine various substances originating from the external environment that have an adverse effect on male fertility. Among the pollutants in the environment that have an effect on the quality of semen, the following can surely be mentioned: phthalates [103], arsenic [104], perfluorinated compounds [105], dimethyl arsenic acid [106], ambient fine particular matter [107], ozone pollution [108], bisphenol A [109], parabens [110], organophosphate pesticides [111], and heavy metals [112]. Unfortunately, these substances are ubiquitous, thus being impossible to fully eliminate. Over the years, several studies have been performed that linked pollutants to insulin sensitivity markers [113,114,115,116]. The strongest evidence for a positive association between exposure to environmental pollutants and insulin resistance was shown in the case of phthalates and air pollutants [115,116].

Other studies suggest that, in addition to pollution, there are other elements of the external environment that may have an effect on fertility. Radwan et al., as well as Zilberlicht et al., showed that carrying a mobile phone in a trouser pocket influences the quality of semen [117,118]. As far as radiation is concerned, it should be noted that radiation therapy, used in cancer treatment, has a negative impact on the quality of semen [119]. Another element that disrupts normal semen production are high temperatures and overheating of testicles, which may occur during long car rides, stays in the sauna, wearing uncomfortable underwear, or due to a sedentary lifestyle [120]. In their long-term study performed in the Chinese population, Zhou et al. showed that both too high and too low ambient temperatures have an effect on the quality of semen, with the optimum temperature quoted at 13 degrees Celsius [121]. As far as weather and atmospheric conditions are concerned, studies indicate seasonal and circadian changes in semen quality—it seems to be in a better condition in the morning; in terms of seasons—in the spring [122,123].

## 4. Conclusions

In conclusion, lifestyle and insulin resistance are both connected to the quality of semen. Thus, men of reproductive age should be educated in the area of the impact of lifestyle and external environment, and the diseases they cause, on the quality of semen and their reproductive health. It should also be noted that the components of lifestyle that have an effect on the quality of semen are connected with insulin resistance. Furthermore, as a lifestyle-related disorder, insulin resistance requires further research in the context of its effect on the quality of semen. In addition, the three factors discussed in this article tend to reappear together in a large number of studies, yet neither a correlation nor a causal relationship between them is indicated in some of those. This suggests that there might exist a substantial knowledge gap in the area of co-dependencies between the three factors in question, which have not yet been studied. Most importantly, performing studies that would compare the quality of semen among insulin resistant and non-insulin resistant men could make it possible to establish a new direction in the diagnostics and treatment of male fertility.

## Data Availability

Not applicable.
